# Sensory Guillain-Barré syndrome: A case report

**DOI:** 10.3892/etm.2014.1995

**Published:** 2014-09-30

**Authors:** JING ZHANG, NA LIU, ZHE-CHENG ZHANG, RUI-ZHI ZHENG, QIAN LI

**Affiliations:** Department of Neurology, Tianjin Third Central Hospital, Tianjin 300170, P.R. China

**Keywords:** sensory Guillain-Barré syndrome, electrophysiology, cranial nerve

## Abstract

A 58-year-old female exhibited the onset of symmetrical sensory abnormalities of the face and extremities. The neurological examination revealed normal muscle strength with abated or absent tendon reflexes. The patient experienced symmetrical glove- and stocking-type pinprick sensations in the distal extremities and a loss of temperature sensation, but had normal proprioception and vibration senses and joint topesthesia. The lumbar puncture showed protein cell separation at the fifth week after the onset of symptoms. At the same time-point, the electrophysiological examination showed demyelination changes involving the trigeminal nerve and the somatic motor nerve. Needle electromyography revealed normal results. The clinical symptoms ceased progression at the fourth week after symptom onset, and began to improve from the sixth. This case was considered to be sensory Guillain-Barré syndrome, which was characterized by its cranial nerve involvement.

## Introduction

Classical Guillain-Barré syndrome (GBS) is a progressive symmetrical limb weakness, in which tendon reflexes disappear. The disease is characterized by an acute onset and the clinical symptoms often reach their peak at the 4th week. GBS is manifested as multiple nerve root and peripheral nerve injury, often with protein-cell separation in the cerebrospinal fluid. It often presents a single-phase self-limiting course; intravenous immunoglobulin and plasma exchange therapy are effective for the treatment of GBS. Demyelination is the main electrophysiological and pathological feature of this disease (1,2). In the past 20 years, it has been recognized that there are extensive subtypes of the condition, which include acute inflammatory demyelinating polyneuropathy, acute motoraxonal neuropathy, acute motor-sensory axonal neuropathy, Miller Fisher syndrome, acute autonomic neuropathy and acute sensory neuropathy. Certain patients with sensory neuropathy may actually exhibit sensory GBS. However, case reports are rare (3,4). A case of sensory GBS treatment is described in the present study.

## Case report

A 58-year-old female patient was admitted to hospital on August 3, 2012 having experienced limb numbness for one month’. This study was conducted in accordance with the Declaration of Helsinki and with approval from the Ethics Committee of the Tianjin Third Central Hospital (Tianjin, China). Written informed consent was obtained from the patient.

One month prior to hospitalization, the patient suddenly felt numbness and pain at the fingertips of the hands accompanied by palpitations. After four days, the symptoms gradually involved the hands and feet and the patient was conscious of lower limb weakness, although this was not accompanied by posture or gait abnormalities. The patient additionally experienced upper gastrointestinal discomfort, but without nausea or vomiting, and zonesthesia from the double costal margin to the umbilical level. After two weeks the patient exhibited numbness of the face, mouth and the skin at the top of the temple. Following symptom onset, she went to the clinic of the Tianjin Dagang Oilfield Hospital (Tianjin, China), where a brain computed tomography (CT) and magnetic resonance imaging examination of the brain and cervical spinal cord showed no abnormalities. The local hospital prescribed Mecobalamine as treatment, but the symptoms continued to progress. During the illness, the patient exhibited no dry eye or dry mouth symptoms, occasionally showed changes in bowel habit (twice a day or once every two days) and exhibited a weight loss of ~6 kg compared with previously. Twenty days previously the patient had also taken a health care product of an unknown name. The patient’s blood glucose levels had increased for two years but, following diet control, her fasting and postprandial blood glucose levels could be maintained at ~7 mmol/l. The patient had no history of habitation in an epidemic or rural environment and no history of smoking or alcohol abuse. She was unaware of any familial hereditary disease history or similar cases in her family.

**Figure 1:**
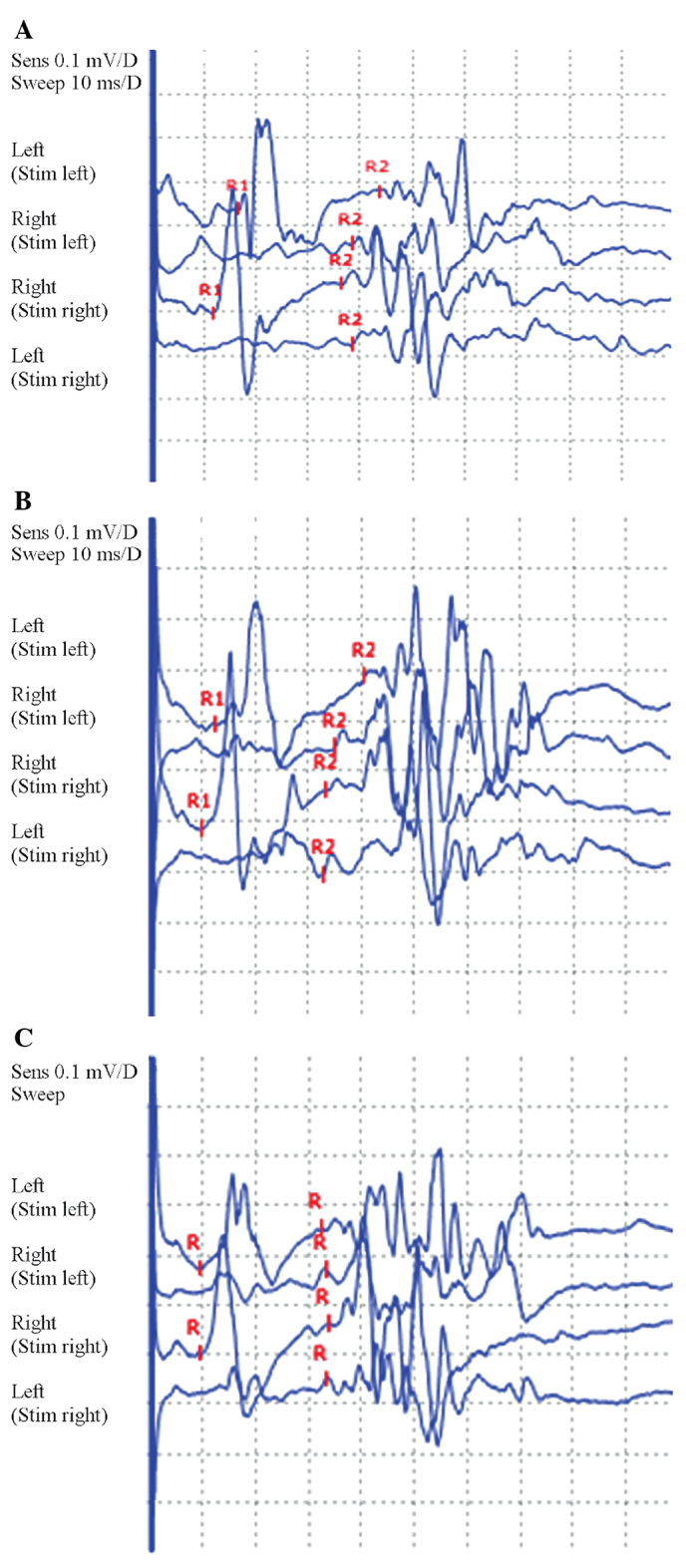
Blink reflex results at different times following the onset. (A) The blink reflex showed ipsilateral R1 and R2 and contralateral R2 latency prolongation at the 4th week after onset. (B) The blink reflex had markedly improved at the 6th week after onset. (C) The blink reflex was approximately normal at the 10th week after onset.

Physical examination on admission indicated the following characteristics: clear and co-operative mentality, with normal advanced neural activity; prefrontal and bilateral facial pain and a loss of heat sensation; no atrophy in the limb muscles; normal muscle tension; muscle strength grade V; positive tendon reflex of the upper limbs, with no tendon reflex of the lower limbs; negative bilateral Hoffmann reflex, with the bilateral Babinski reflex not being elicited; normal gait and no ataxia. The patient experienced glove- and stocking-type sensations in her hands and feet and a loss of pain and temperature sensation. The patient’s diapason vibration sensation and joint position sense were normal. In addition, her discriminative touch sense was regular, and the internal medical examination revealed no abnormalities. A routine blood test was conducted following admission, as well as tests for liver and kidney function, five types of hepatitis B, syphilis, human immunodeficiency virus, thyroid function, tumor markers, immune components, vitamin B_12_, folic acid, fasting blood glucose, mercury, lead, manganese, chromium and other toxins. All results were normal. A gastroscopy, chest CT, abdominal B-ultrasound, echocardiography and thyroid B-ultrasound showed no evident abnormalities. On the second day after admission, the electrophysiological examination was performed. The results revealed that the bilateral median, ulnar, right posterior tibial and peroneal nerves exhibited prolonged distal motor latency, the amplitude was reduced, the proximal amplitude was reduced with a normal speed, and the distal sensory nerve did not elicit a positive waveform. The results of the needle electromyography of the abductor digiti minimi and anterior tibial muscles were normal. Following stimulation of the bilateral median, ulnar and posterior tibial nerves there was a normal F wave, and following stimulation of the bilateral posterior tibial nerve there was no H reflex. In addition, the blink reflex showed prolongation of the ipsilateral R1 and R2 and contralateral R2 latency. Finally, the facial nerve motor conduction was normal, suggesting that the damage may have been to the trigeminal primary afferent (Tables I–III and Fig. 1A). Examination of the cerebrospinal fluid showed the number of cells and glucose and chloride levels to be normal, while the protein levels were increased to 131.7 mg/dl and the oligoclonal band was negative. Following admission, the patient was diagnosed with sensory GBS, and was administered γ globulin at a dosage of 400 mg/kg/day, intravenous immunoglobulin, for five consecutive days, and heteropathy with vitamins B_1_ and B_12_, and neurotropin. On August 16 (six weeks after the symptom onset), the review of the neurophysiology showed that the peripheral nerve motor conduction amplitude had recovered, and there were no clear changes in the motor distal latency and sensory conduction results. The needle electromyography results of the right abductor digiti minimi and anterior tibial muscles showed much denervation potential. The blink reflex was significantly improved (Tables I–III and Fig. 1B). The review of the lumbar puncture showed protein levels to be 96.8 mg/dl on August 17. On August 18, the clinical symptoms had completely remitted, and the patient was discharged. Ten weeks after the onset of symptoms, the review of the neurophysiological results showed that the amplitude of the peripheral nerve motor conduction had further recovered and the distal latency had improved. The sensory conduction results had not changed significantly. The abductor digiti minimi and anterior tibial muscles needle electromyography results returned to normal, and the blink reflex was approximately normal (Tables I–III and Fig. 1C).

## Discussion

The clinical features exhibited by the patient included numbness of the extremities, accompanied by the abatement and disappearance of tendon reflexes and subjective fatigue. Objective examination revealed muscle strength to be normal. Symptoms reached their peak in four weeks. Analysis of cerebrospinal fluid showed protein cell separation and the electrophysiological examination showed primarily distal sensorimotor fiber demyelination changes. Following treatment, the clinical symptoms were alleviated from the sixth week after symptom onset, and the protein levels in the cerebrospinal fluid reduced from the levels of the fourth week. The clinical and laboratory characteristics were consistent with classic GBS (5).

Oh *et al* (6) proposed nine criteria for the diagnosis of sensory GBS in 2001: i) Acute symmetrical sensory loss, ii) a peak in symptoms at four weeks, iii) abating or disappearing tendon reflexes, iv) normal muscle strength, v) at least two pieces of evidence for nerve demyelination in the electrophysiological examination, vi) single-phase course, vii) the exclusion of other neurological diseases, viii) no family history, and ix) increases in protein levels in the cerebrospinal fluid in the acute phase. As described, the patient met all the aforementioned diagnostic criteria. However, clinical case reports about sensory GBS remain rare, and the understanding of this type of sensory GBS remains superficial.

Firstly, clinical and electrophysiological characteristics of sensory GBS show heterogeneity. Seneviratne and Gunasekera (7) reported six cases of sensory GBS with clinical manifestations of sensory impairment to the extremities but no deep sensory abnormalities or ataxia. The electrophysiological examinations were normal, and cerebrospinal fluid examination showed isolated protein cells. The six patients had a good prognosis, considering the effects of the small fiber damage. Dawson *et al* (8) described a case of sensory GBS in which the patient exhibited abnormal sensation and joint position sense, vibratory sensory abnormalities and ataxia. Certain patients may exhibit subjective weak limb muscle strength, and electrophysiology tests can show demyelination of the involved motor fiber, which is also considered as a lesion in the large sensory fiber. Lee and Lee (9) believed that those patients who showed only clinical sensory neuropathy, and who were indicated to have motor and sensory fiber demyelination by electrophysiological examination, or demyelination only involving the sensory fibers, could be diagnosed with sensory type GBS.

In view of the clinical and electrophysiological characteristic heterogeneity of sensory GBS, Uncini and Yuki (10) suggested that, according to the initial injury site and the diameter of the involved fibers, sensory GBS could be divided into three categories: Acute sensory demyelinating polyneuropathy, acute sensory large-fiber axonopathy-ganglionopathy and acute sensory small-fiber neuropathy-ganglionopathy. In the present case, the clinical manifestations in the patient were sensory disturbance and mild fatigue. The objective examination did not reveal loss of muscle strength; however, the electrophysiological examination suggested evidence of sensorimotor fiber demyelination; this was considered to be a type of acute sensory fiber demyelinating polyneuropathy. This type of sensory GBS is very similar to classical acute inflammatory demyelinating polyneuropathy (AIDP); however, the difference is primarily sensory injury. Of note, the patient exhibited clinical manifestations of trigeminal nerve involvement, and electrophysiology tests provided objective evidence of trigeminal nerve involvement, which has not been reported in previous cases. It is therefore confirmed that there can be sensory cranial nerve fiber involvement in cases of sensory GBS, just as AIDP can have motor cranial nerve fiber involvement. There are also numerous sensory neuropathies with acute or subacute sensory disturbances as the clinical onset, and the differential points between these and sensory GBS remain unclear. Yee and Katz (11) proposed that sensory disturbance primarily exhibits a multi-asymmetric onset, but this feature is not specific; the truly significant differential feature is that the former exhibits continuous clinical symptoms, and the latter is a one-way course. However, it can be observed from the present case that while sensory disturbances only involve sensory fibers, sensory GBS can exhibit the clinical involvement of motor fibers. Furthermore, the one-way course of sensory GBS indicates a clinical cure, but the electrophysiological examination manifests the abnormality. For patients in the present study, the clinical symptoms ceased, but the electrophysiological examination did not reveal complete restoration, which is consistent with the studies by Bannister and Sears (12) and Sauron *et al* (13). It has been suggested that myasthenia can no longer be considered to be the core symptom, while a prodrome of GBS and cerebrospinal fluid protein cell separation are not required conditions for the diagnosis of GBS.

In conclusion, further understanding of the features of sensory GBS would be greatly advantageous for improving the treatment rate and enabling patients with GBS to receive timely and effective treatment. Sensory GBS is an important type of GBS, which may be associated with involvement of the cranial nerve.

## Figures and Tables

**Table I:** Right median and ulnar nerve motor conduction results at different times after onset.

	Median nerve			Ulnar nerve		
		
Time after onset (weeks)	Distal latency (msec, % change)	Amplitude (mV, % change)	Speed, elbow-wrist (m/sec)	Distal latency (msec, % change)	Amplitude (mV, % change)	Speed, elbow-wrist (m/sec)
4	15.2, ↑347	1.6, ↓89	52.6	4.27, ↑64	4.3, ↓75	67.1
6	15.5, ↑356	4.1, ↓74	57.1	4.53, ↑74	7.6	70.1
10	9.7, ↑185	4.7, ↓71	53.8	3.30, ↑27	7.8	70.4

% change: ↑, increase; ↓decrease; no label, normal values.

**Table II:** Right median and ulnar sensory nerve sensory conduction results at different times after onset.

	Median nerve				Ulnar nerve			
		
	Distal (finger 1-wrist)				Proximal (wrist-elbow)			
		
Time after onset (weeks)	Amplitude (μV)	Speed (m/sec)	Amplitude (μV)	Speed (m/sec)	Amplitude (μV, % change)	Speed (m/sec)	Amplitude (μV)	Speed (m/sec)
4	No	No	22.7	69.2	No	No	13.3	66.7
6	No	No	25.0	65.5	No	No	11.4	70.7
10	No	No	24.8	66.8	2.7, ↓86%	48.8	12.8	68.9

‘No’, no positive waveform elicited; no label, normal values.

**Table III:** Needle electromyography results of the patient at different times after onset.

Muscle	Time after onset (weeks)	Rest	Minimal contraction (MUP)		Maximal contraction (interference phase, mV)
	
		Denervation potential	Duration (msec)	Amplitude (μV)	
Right abductor minimi muscle					
	4	(−)	12.9	478	2.87
	6	P++++F++	11.9	498	2.89
	10	(−)	12.6	793	3.52
Right anterior tibial muscle					
	4	(−)	13.7	544	3.13
	6	P++++F++	11.6	529	3.08
	10	(−)	11.8	580	3.46

P, positive wave; F, fibrillation potential; MUP, motor unit potential; ++++, large denervation potential; ++, moderate denervation potential.
